# The Importance of Advanced Imaging in Diagnosing and Differentiating Lung Carcinoid Tumors From Pulmonary TB and Upper Respiratory Infections

**DOI:** 10.7759/cureus.72158

**Published:** 2024-10-22

**Authors:** Norah H Aung, Wut Y Hlaing, Hla Thwe, Htat A Aung

**Affiliations:** 1 Internal Medicine, University of Medicine 1, Yangon, MMR; 2 Internal Medicine, Harlem Hospital Center, Macomb, USA; 3 Public Health, Western Illinois University, Macomb, USA; 4 Sleep Medicine, Hennepin Healthcare, Minneapolis, USA; 5 Internal Medicine, New York City Health and Hospitals Corporation/Harlem, New York City, USA; 6 Internal Medicine, Richmond University Medical Center, Staten Island, USA

**Keywords:** advanced imaging, ct scan, differential diagnosis, hemoptysis, lung carcinoid tumor, pet/ct, pulmonary tuberculosis, upper respiratory infection

## Abstract

Lung carcinoid tumors, pulmonary tuberculosis (TB), and upper respiratory infections (URIs) present with overlapping symptoms, making accurate diagnosis challenging. This case report illustrates the diagnostic difficulties and the critical role of advanced imaging in distinguishing these conditions. We describe a 58-year-old Southeast Asian non-smoker woman with a history of pulmonary TB who presented with intermittent hemoptysis over four months. Initial treatments for URIs and TB, including multiple courses of antibiotics and chest X-rays, did not alleviate her symptoms. Advanced imaging with a CT scan revealed a 3 × 3 cm nodule in the retrocardiac area of the left lower lobe, leading to a diagnosis of a lung carcinoid tumor following biopsy. The patient underwent a left lower lobe lobectomy, which resulted in significant clinical improvement. This case highlights the limitations of initial diagnostic approaches and underscores the importance of advanced imaging techniques such as CT and PET/CT in accurately identifying and characterizing lung tumors. These modalities are essential not only for diagnosing but also for guiding appropriate treatment and improving patient outcomes. Advanced imaging allows for precise differentiation between lung carcinoid tumors, TB, and URIs, ensuring that patients receive timely and effective management. The findings of this case reinforce the need for comprehensive imaging in the evaluation of persistent respiratory symptoms, advocating for the integration of advanced imaging into routine clinical practice to enhance diagnostic accuracy and treatment efficacy.

## Introduction

Lung carcinoid tumors, pulmonary tuberculosis (TB), and upper respiratory infections (URIs) are distinct clinical entities with overlapping symptoms, posing significant diagnostic challenges. Lung carcinoid tumors are rare neuroendocrine neoplasms, representing approximately 1-2% of all lung cancers [1,2]. They are classified into typical and atypical subtypes based on histological features, with typical carcinoids having fewer mitoses and lacking necrosis, while atypical carcinoids demonstrate higher mitotic rates and necrosis [1]. Clinically, lung carcinoids may present with hemoptysis, cough, and recurrent pulmonary infections [2]. Pulmonary TB, caused by *Mycobacterium tuberculosis*, remains a global health concern, especially in developing countries. It primarily affects the lungs, causing chronic cough, hemoptysis, night sweats, weight loss, and fever. The diagnosis of TB relies on clinical evaluation, radiographic imaging, and microbiological confirmation [3]. Radiographic features of TB can include consolidation, cavitation, fibrosis, and nodular lesions, which can complicate differentiation from lung carcinoma or carcinoid tumors [4].

URIs include conditions such as the common cold, pharyngitis, and laryngitis, mainly caused by viruses, presenting with sore throat, nasal congestion, cough, and sometimes fever. Chronic or recurrent URIs can lead to diagnostic dilemmas when presenting with persistent cough and hemoptysis [5]. The intersection of these conditions highlights the importance of advanced imaging techniques like CT and PET scans for accurate diagnosis, guiding appropriate management, and improving patient outcomes [6,7]. This case report underscores the need for advanced imaging in differentiating these conditions due to their overlapping symptoms and varied management approaches.

## Case presentation

**Figure 1:**
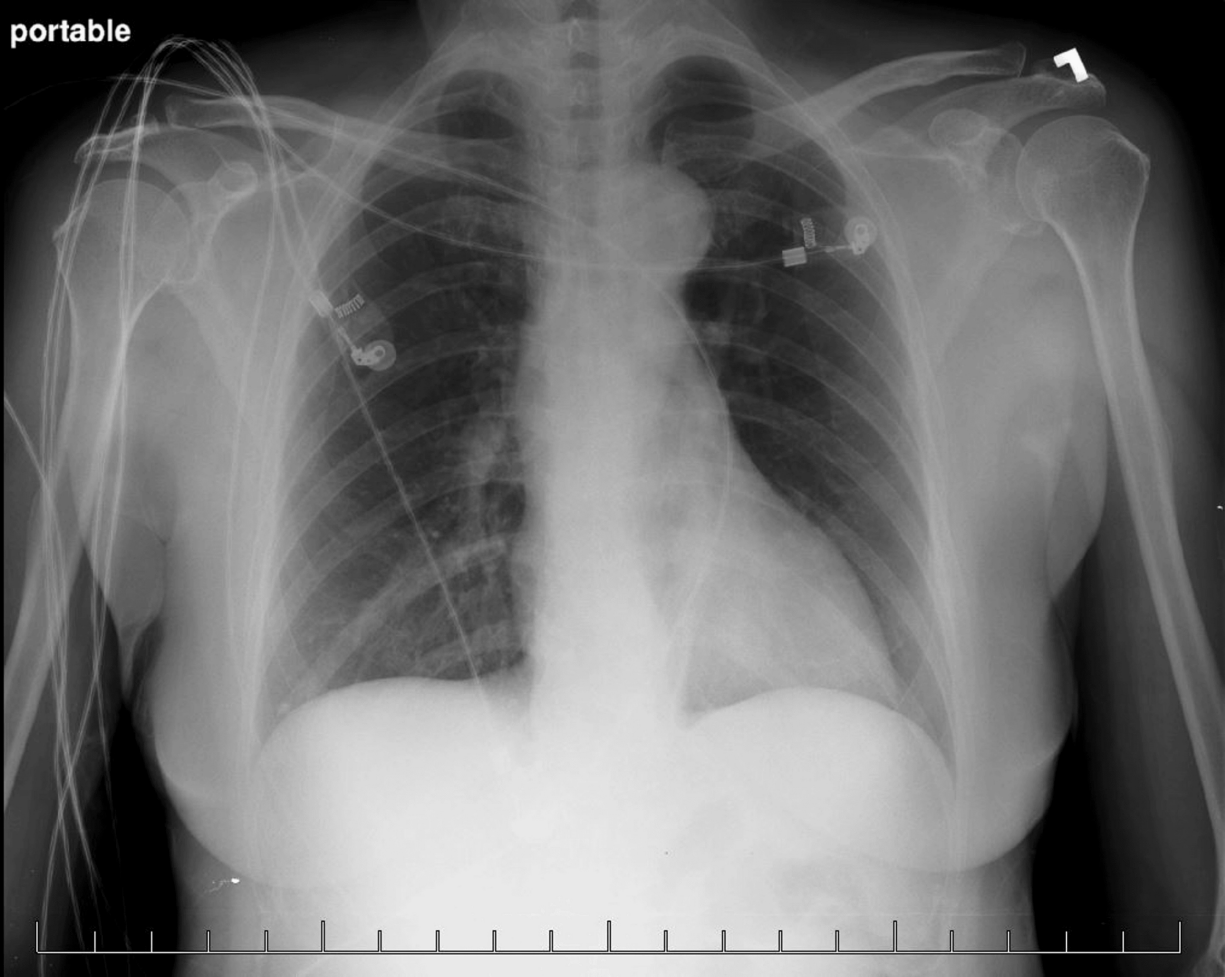
Chest X-ray in AP showed a possible infiltrate on the middle and lower lobes of the right lung.

A 58-year-old Southeast Asian non-smoker woman presented with a four-month history of intermittent hemoptysis with a previous history of primary TB. She contracted TB four years ago, for which she completed the Rifampin, Isoniazid, Pyrazinamide, and Ethambutol (RIPE) TB treatment for six months in total with four drug regimens for two months and two drug regimens for four months. In the initial month of the presentation, she noticed some bright color blood streaks mixed with whitish sputum, especially in the morning. She did not have any blood streaks throughout the day. Thus, she thought her body was just tired, and she might have some respiratory infection. She denies fever, chills, night sweats, weight loss, or sick contact. She was seen by a primary care doctor. A chest X-ray in anteroposterior (AP) view, as shown in Figure 1, was obtained, which showed a possible infiltration on the right middle and lower lobe. She was then prescribed a 10-day course of oral Azithromycin for possible upper respiratory tract infection. After completing a course of antibiotics, she neglected her symptoms as the symptoms improved initially.

**Figure 2:**
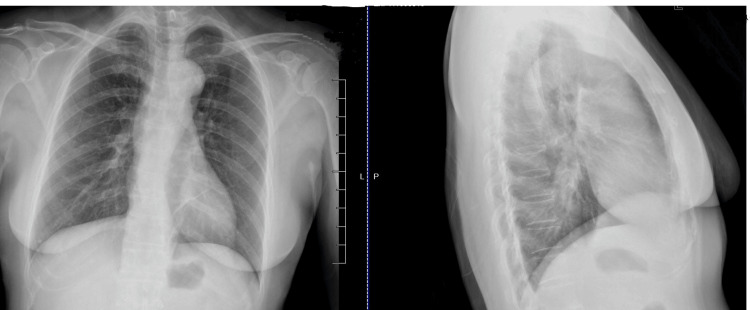
Chest X-rays in PA and lateral view showed no infiltrate, no tumor, or no scar.

After about four months, her hemoptysis came back and got worse. At that time, she was coughing with brown blood clots in the early morning. She was again seen by a primary care doctor. Another chest X-ray was obtained, and the findings suggested an upper respiratory tract infection, and she started on Oral Azithromycin for 10 days. Despite the first antibiotic course treatment, her symptoms did not improve. Thus, she was prescribed Oral Augmentin (amoxicillin and clavulanate) with close follow-up visits and sequential chest X-rays in posterior-anterior (PA) view and lateral view, as shown in Figure 2 did not show any infiltrate, any tumor, or any scar. Her symptoms still did not improve, and she was then referred to a pulmonologist to evaluate for not improving symptoms. She was then prescribed oral Levofloxacin, but at that time, the duration of the course was extended to two weeks. Despite the extended course of Levofloxacin, her symptoms did not improve. A few more chest X-rays were repeated in different locations, but no significant findings were found. Thus, her pulmonologist started RIPE TB treatment as she has a previous history of pulmonary TB, which can be a case of reactivation of pulmonary TB in developing countries from Southeast Asia. Three separate acid-fast bacilli (AFB) cultures came back negative. There was no obvious progress even after completing the full course of recommended different antibiotic regimes and RIPE TB treatment.

**Figure 3:**
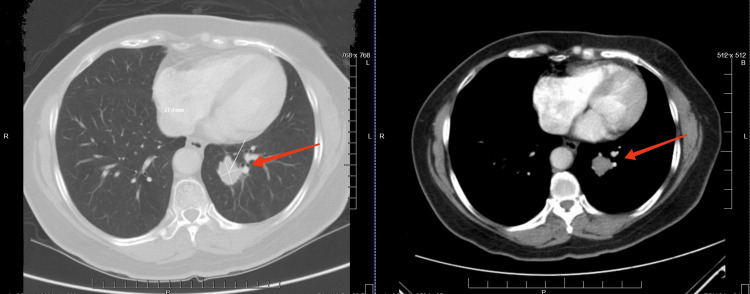
Chest CT with contrast showed a ~3 × ~3 cm nodule in the retrocardiac area in the lower lobe of the left lung.

Due to the unlikelihood of pulmonary TB reactivation and upper respiratory tract infection, a CT scan of the chest with contrast was finally obtained. CT chest with contrast in Figure 3 showed a ~3 × ~3 cm nodule in the retrocardiac area in the lower lobe of the left lung. All the above clinical courses occurred in Myanmar (former name: Burma). As she is a United States (US) immigrant and her daughters reside in the US, she migrated to New York City, where her daughters reside. She was then subsequently admitted to the hospital. She was evaluated by thoracic surgery and hemato-oncology teams. With the collaboration of teamwork evaluation, left lower lobe lobectomy was done after careful consideration in management. Her lung nodule biopsy resulted in a carcinoid tumor of the lungs. She slowly recovered eventually.

## Discussion

Differentiating between lung carcinoid tumors, TB, and URIs based on clinical presentation alone presents significant challenges due to overlapping symptoms and non-specific findings. Lung carcinoid tumors, though rare, can manifest with symptoms such as intermittent hemoptysis, cough, and recurrent respiratory infections, which are also common in TB and URIs. Hemoptysis is a hallmark of both lung carcinoid tumors and TB, often leading to initial misdiagnosis or delayed diagnosis [1,2]. In regions with high TB prevalence, hemoptysis and persistent cough are frequently attributed to TB, especially in patients with a history of the disease, delaying the identification of lung carcinoid tumors [3,8]. URIs, typically viral, present with symptoms such as sore throat, nasal congestion, and cough, often leading to conservative management with antibiotics, complicating the diagnostic process when symptoms persist [5].

Initial diagnostic tools like chest X-rays may not reveal small or centrally located tumors and can be inconclusive in differentiating TB reactivation from neoplastic processes, necessitating more advanced imaging modalities for definitive diagnoses [4,7]. Advanced diagnostic techniques, such as CT and PET scans, offer detailed anatomical and functional insights crucial for distinguishing between these conditions and guiding appropriate and timely therapeutic interventions [6,9]. Lung carcinoid tumors typically appear on CT scans as well-defined, round, or ovoid nodules or masses with intense enhancement due to their vascular nature [1,7]. PET scans show increased radiotracer uptake, indicating hypermetabolic activity characteristic of these tumors [9]. In contrast, pulmonary TB can manifest a wide range of radiographic patterns, including consolidation, cavitation, fibrosis, and nodular opacities, often located in the upper lobes. Cavitary lesions with thick walls and surrounding nodularity are particularly suggestive of active TB [4,8]. URI complications can show bronchial wall thickening, peribronchial cuffing, and patchy areas of consolidation on CT scans [5].

MRI provides additional detail, particularly in assessing soft tissue involvement and differentiating tumor tissue from surrounding structures, and is beneficial when other imaging findings are inconclusive [10]. Overall, CT and PET scans are essential for identifying and characterizing lung carcinoid tumors, while the diverse imaging features of pulmonary TB and URIs highlight the need for a comprehensive diagnostic approach [6,11]. The case of a 58-year-old Southeast Asian non-smoker woman with intermittent hemoptysis and a history of pulmonary TB aligns with existing literature on the diagnostic challenges and importance of advanced imaging in differentiating between lung carcinoid tumors, TB, and URIs. This patient presented with recurrent respiratory symptoms initially attributed to possible URIs and reactivation of TB. Despite multiple courses of antibiotics and negative AFB cultures, advanced imaging with a CT scan eventually revealed a lung carcinoid tumor, underscoring the necessity of detailed imaging in cases of diagnostic uncertainty [4,7].

## Conclusions

This case of a 58-year-old Southeast Asian woman with a history of TB and intermittent hemoptysis highlights the diagnostic challenges in distinguishing lung carcinoid tumors from TB and URIs. Despite multiple antibiotic courses, her symptoms persisted until a CT scan revealed a 3 × 3 cm nodule, leading to the diagnosis of a lung carcinoid tumor. This underscores the limitations of conventional diagnostics and the vital role of advanced imaging.

Advanced imaging modalities like CT, PET/CT, and MRI are essential for accurate diagnosis and management by providing detailed anatomical and metabolic information. These techniques facilitated an accurate diagnosis and effective surgical intervention in this case, significantly improving the patient’s prognosis. In clinical practice, advanced imaging ensures timely and appropriate care, preventing misdiagnosis and unnecessary treatments. Clinicians should promptly use advanced imaging for persistent respiratory symptoms with inconclusive initial findings to explore significant diagnoses such as lung carcinoid tumors, leading to better patient outcomes.
